# Convergent synthesis and evaluation of ^18^F-labeled azulenic COX2 probes for cancer imaging

**DOI:** 10.3389/fonc.2012.00207

**Published:** 2013-01-03

**Authors:** Donald D. Nolting, Michael Nickels, Mohammed N. Tantawy, James Y. H. Yu, Jingping Xie, Todd E. Peterson, Brenda C. Crews, Larry Marnett, John C. Gore, Wellington Pham

**Affiliations:** ^1^Department of Radiology, Institute of Imaging Science, Vanderbilt UniversityNashville, TN, USA; ^2^Department of Physics and Astronomy, Vanderbilt UniversityNashville, TN, USA; ^3^Department of Chemistry, Vanderbilt UniversityNashville, TN, USA; ^4^Vanderbilt Institute of Chemical BiologyNashville, TN, USA; ^5^Vanderbilt Ingram Cancer Center, Vanderbilt UniversityNashville, TN, USA; ^6^Department of Biomedical Engineering, Vanderbilt UniversityNashville, TN, USA; ^7^Department of Molecular Physiology and Biophysics, Vanderbilt UniversityNashville, TN, USA; ^8^Department of Neuroscience, Vanderbilt UniversityNashville, TN, USA

**Keywords:** azulene, COX2, breast cancer, PET, CT, convergence synthesis, molecular imaging

## Abstract

The overall objectives of this research are to (i) develop azulene-based positron emission tomography (PET) probes and (ii) image COX2 as a potential biomarker of breast cancer. Several lines of research have demonstrated that COX2 is overexpressed in breast cancer and that its presence correlates with poor prognoses. While other studies have reported that COX2 inhibition can be modulated and used beneficially as a chemopreventive strategy in cancer, no viable mechanism for achieving that approach has yet been developed. This shortfall could be circumvented through *in vivo* imaging of COX2 activity, particularly using sensitive imaging techniques such as PET. Toward that goal, our laboratory focuses on the development of novel ^18^F-labled COX2 probes. We began the synthesis of the probes by transforming tropolone into a lactone, which was subjected to an [8 + 2] cycloaddition reaction to yield 2-methylazulene as the core ring of the probe. After exploring numerous synthetic routes, the final target molecule and precursor PET compounds were prepared successfully using convergent synthesis. Conventional ^18^F labeling methods caused precursor decomposition, which prompted us to hypothesize that the acidic protons of the methylene moiety between the azulene and thiazole rings were readily abstracted by a strong base such as potassium carbonate. Ultimately, this caused the precursors to disintegrate. This observation was supported after successfully using an ^18^F labeling strategy that employed a much milder phosphate buffer. The ^18^F-labeled COX2 probe was tested in a breast cancer xenograft mouse model. The data obtained via successive whole-body PET/CT scans indicated probe accumulation and retention in the tumor. Overall, the probe was stable *in vivo* and no defluorination was observed. A biodistribution study and Western blot analysis corroborate with the imaging data. In conclusion, this novel COX2 PET probe was shown to be a promising agent for cancer imaging and deserves further investigation.

## INTRODUCTION

Prostaglandin endoperoxide synthase, known more commonly as cyclooxygenase (COX), is the key enzyme required for the conversion of arachidonic acid to the biological mediators known as prostanoids, which include prostaglandins, prostacyclin, and thromboxane (Moore and Simmons, 2000). The two COX isoforms, COX1 and COX2, are expressed in different tissue at varying degrees (Dubois et al., 1998). While COX1 is expressed under basal conditions in almost all tissues and is particularly important to the maintenance of gastric mucosal integrity, renal function, and hemostasis, COX2 is undetectable in most normal tissues (van Ryn et al., 2000). COX2 is highly inducible in cells involved in inflammation and cancer (Rouzer and Marnett, 2009). In addition to the role it plays in inflammation, several lines of research suggest that COX2 is involved in the early stage of tumorigenesis (Yokota et al., 1986; Xie et al., 1991). Notably, COX2 not only continues to express during tumor progress, but the expression of COX2 also indicates an aggressive tumor phenotype that behaves more invasively (Fujita et al., 1998) and thus, a poor prognosis (Sobolewski et al., 2010). COX2 overexpression has been well documented in several human carcinomas including colon (Nasir et al., 2011), stomach (Murata et al., 1999), lung (Hida et al., 1998), breast (Glynn et al., 2010; Singh et al., 2011), head and neck (Chan et al., 1998), bladder (Shimada et al., 2011), and pancreas (Hill et al., 2012).

The relationship between cancers and increased COX2 activity provides a rationale for the use of COX2 as a prognostic marker and as a quantifiable indicator of tumor progression and treatment efficacy. Collectively, this approach could be achieved through *in vivo* imaging of COX2 activity, especially when using a sensitive imaging technique such as positron emission tomography (PET). A number of research initiatives have reported the development of COX2 probes with which to visualize cancer-related inflammation including its use in optical (Uddin et al., 2010) and PET imaging (McCarthy et al., 2002; Prabhakaran et al., 2005; Uddin et al., 2011). Our laboratory has focused on the development of azulene-based COX2 probes owing to the nanomolar affinity and high selectivity toward the COX2 enzyme reported previously (Tomiyama et al., 1999). Azulene has a structural backbone similar to indomethacin and sulindac, two of the most common non-steroidal anti-inflammatory drugs (NSAIDs). However, the difference between such NSAIDs and this non-benzenoid aromatic hydrocarbon is the existence of a 7-member ring. According to Tomiyama et al. (1999) azulene is suitable for COX2 development since the larger ring fits well within the larger binding pocket of COX2 compared to COX1, which enhances COX2 selectivity.

**FIGURE 1:**
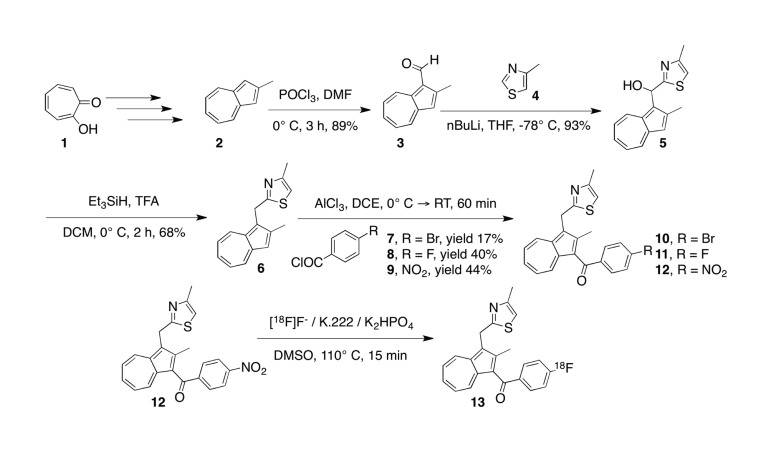
**The design of a convergent synthesis approach to develop an azulene-based ^18^F-COX2 probe 13 and its related precursors**.

Herein, we describe a novel chemistry approach that uses a convergent synthesis methodology to develop azulene-based COX2 PET probes. Of note, we synthesized the main azulene ring using the procedure we reported previously (Pham et al., 2002; Nolting et al., 2009). The two other ring structures were assembled onto the azulene ring using commercially available analogs. To retain the biological activity as reported by Tomiyama et al. (1999), we designed the precursors specifically with ^18^F fluoride labeling in mind. Not only do we prefer this isotope due to its relatively long half-life, but also because replacing a hydrogen atom with a fluorine is likely to not affect biological activity since they are very similar sterically (Jalilian et al., 2000; Mueller et al., 2007). We also report herein, to our knowledge, the first time, a modified labeling condition that uses dipotassium phosphate (K_2_HPO_4_) for this family of compounds, which we found to be unstable using the conventional PET labeling process. Overall, the chemical yield of this 7-step synthesis of the nitro precursor **12** (**Figure 1**) is 25%. The biodistribution results and small animal PET imaging demonstrate the potential use of the ^18^F-COX2 probe in breast cancer imaging.

## MATERIALS AND METHODS

### CHEMICALS AND CHARACTERIZATION

We synthesized 2-methyl azulene **2** and reported that outcome in previous publications (Pham et al., 2002; Nolting et al., 2009). All reagents were obtained through commercial sources such as Sigma–Aldrich, Acros, or Tokyo Chemical Industry (TCI) and were used without further purification. Solvents were purified using the PureSolv MD purification system. All reactions were conducted in argon-flushed, rubber septum-sealed flasks, and the reagents were introduced via tight-gas syringes. Reaction progress was monitored by thin layer chromatography (TLC) on pre-coated silica gel plates. Visualization was accomplished by the naked eyes and by 254 nm-UV light. Flash chromatography separations were performed using Biotage and Teledyne systems. HPLC analysis and purification were performed using diode array Hitachi LaChrome Elite® systems. ^1^H NMR and ^13^C NMR spectra were recorded on a Bruker 400 MHz spectrometer in CDCl_3_ using tetramethylsilane (TMS) as the internal standard. All chemical shifts were reported in ppm.

#### 2-Methylazulene-1-carbaldehyde (Compound **3**)

POCl_3_ (3.86 mL, 42.2 mmol) was added slowly to a stirring dimethylformamide (DMF) solution at 0°C. The mixture was cooled for 1 h before a DMF solution of 2-methyl azulene (2.0 g, 14.1 mmol) was added drop-wise. The reaction was mixed for 2 h at 0°C, and then quenched with cold 10% NaOH. The organic layer was extracted into ethyl acetate after which the extracts were washed with water and brine, dried with MgSO_4_ and purified using flash chromatography (ethyl acetate and hexane). The purified material was dried down into a dark red solid. Yield: 2.389 g, 89%. ^1^H NMR (400 MHz, CDCl_3_) *δ* 10.50 (s, 1 H), 9.44 (d, *J* = 9.73 Hz, 1 H), 8.29 (d, *J* = 9.72 Hz, 1 H), 7.72 (t, *J* = 9.82 Hz, 1 H), 7.56 (t, *J* = 9.82 Hz, 1 H), 7.46 (t, *J* = 9.67 Hz, 1 H), 7.08 (s, 1 H), 2.84 (s, 3 H); ^13^C NMR (100 MHz, CDCl_3_) *δ* 185.8, 154.9, 144.4, 142.1, 137.9, 136.4, 129.7, 128.5, 122.5, 120.3, 15.1. HRMS (ES) calcd. MH^+^ (C_12_H_11_O) 171.0732 found 171.0804.

#### (2-Methylazulen-1-yl)(4-methylthiazol-2-yl)methanol (compound **5**)

4-Methyl thiazole **4** (837 μL, 9.2 mmol) was added to 20 mL of tetrahydrofuran (THF). The mixture was cooled to -78°C and stirred for 20 min. Afterward, 2.5 M nBuLi (2.45 mL, 6.1 mmol) was added slowly over the course of 15 min. The resultant mixture was stirred at -78°C for 1 h, after which a freshly made solution of 2-methylazulene-1-carbaldehyde **3** (522 mg, 3.1 mmol) in THF was added slowly at -78°C. The reaction was stirred for 30 min and checked by TLC (50:50 Hexanes/EtOAc). Hexane was added at -78°C and the reaction was warmed to room temperature. Water was added to quench the reaction and the organic layer was extracted into ethyl acetate. The extracts were washed with water and brine, dried with MgSO_4_ and purified using flash chromatography (ethyl acetate and hexane). The purified material was dried down into a purple solid. Yield: 768 mg, 93%. ^1^H NMR (400 MHz, CDCl_3_) *δ* 8.45 (d, *J* = 9.85 Hz, 1 H), 8.19 (d, *J* = 9.54 Hz, 1 H), 7.52 (t, *J* = 9.82 Hz, 1 H), 7.19–7.13 (m, 3 H), 6.78 (s, 1 H), 6.64 (s, 1 H), 2.56 (s, 3 H), 2.42 (s, 3 H); ^13^C NMR (100 MHz, CDCl_3_) *δ* 174.2, 152.2, 149.1, 140.6, 137.2, 136.1, 134.8, 132.4, 125.3, 123.8, 123.6, 118.7, 114.1, 67.8, 16.9, 15.6. HRMS (ES) calcd. [M-H_2_O+H]^+^ (C_16_H_14_NS) 252.0925, found 252.0836.

#### 4-Methyl-2-((2-methylazulen-1-yl)methyl)thiazole (compound **6**)

Triethylsilane (178 μL, 1.11 mmol) was added slowly to 2 mL of trifluoroacetic acid (TFA) at room temperature. The mixture was cooled to 0°C and mixed for 30 min. A fresh solution of (2-methylazulen-1-yl)(4-methylthiazol-2-yl)methanol **5** (100 mg, 0.371 mmol) in dichloromethane was then added slowly to the mixture being stirred at 0°C. The reaction was kept at 0°C for 2 h and then warmed to room temperature. Afterward, the mixture was poured into cold 20% KOH to quench the reaction. The organic layer was extracted into diethyl ether and washed with water and brine, dried with MgSO_4_ and purified using flash chromatography (ethyl acetate and hexane). The purified material was dried down into a blue solid. Yield: 64.3 mg, 68%. ^1^H NMR (400 MHz, CDCl_3_) *δ* 8.19 (t, *J* = 10.38 Hz, 2 H), 7.49 (t, *J* = 9.90 Hz, 1 H), 7.21 (s, 1 H), 7.17–7.10 (m, 2 H), 6.60 (s, 1 H), 4.68 (s, 2 H), 2.60 (s, 3 H), 2.42 (s, 3 H); ^13^C NMR (100 MHz, CDCl_3_) *δ* 171.8, 152.1, 149.0, 140.1, 137.0, 135.8, 134.4, 131.5, 123.2, 122.7, 117.8, 113.1, 29.3, 17.0, 15.0. HRMS (ES) calcd. MH^+^ [C_16_H_16_NS]^+^ 254.0925, found 254.0990.

#### (2-Methyl-3-((4-methylthiazol-2-yl)methyl)Azulen-1-yl)(4-nitrophenyl)methanone(compound **12**)

AlCl_3 _(101 mg, 0.757 mmol) was weighed quickly into an argon-flushed vial. While the vial was being purged with argon, dichloroethane was added slowly. The ensuing mixture was syringed quickly into a round-bottom flask and cooled to 0°C. A solution of 4-nitro benzoyl chloride (70 mg, 0.377 mmol) in dichloroethane was added slowly into the suspension of AlCl_3_ at 0°C. This mixture was stirred at 0°C for 30 min after which a fresh solution of compound **6** (64 mg, 0.253 mmol) in dichloroethane was added slowly to the reaction mixture being stirred at 0°C. After the reaction was stirred at 0°C for 30 min, it was brought to room temperature and then stirred for another 30 min. The reaction was quenched by adding ice-cold water slowly. The organic layer was extracted into dichloromethane and washed with water and brine, dried with MgSO_4_ and purified using flash chromatography (ethyl acetate and hexane). The purified material was dried down into a brown/orange solid. Yield: 45 mg, 44%. ^1^H NMR (400 MHz, CDCl_3_) δ 8.63 (d, *J* = 9.84 Hz, 1 H), 8.44 (d, *J* = 9.89 Hz, 1 H), 8.29 (d, *J* = 8.82 Hz, 2 H), 7.86 (d, *J* = 8.81 Hz, 2 H), 7.71 (t, *J* = 9.82 Hz, 1 H), 7.46 (t, *J* = 9.72 Hz, 1 H), 7.29 (t, *J* = 9.93 Hz, 1 H), 6.67 (s, 1 H), 4.70 (s, 2 H), 2.42 (s, 3 H), 2.39 (s, 3 H); ^13^C NMR (100 MHz, CDCl_3_) δ 192.2, 170.3, 152.3, 150.4, 149.5, 146.3, 141.4, 140.2, 138.2, 135.6, 133.7, 130.2, 127.2, 125.0, 124.3, 123.6, 123.3, 113.2, 29.0, 16.9. HRMS (ES) calcd MH^+^ [C_23_H_19_N_2_O_3_S]^+^403.1038, found 403.1106.

### LABELING SYNTHESIS

No-carrier-added [^18^F]F^-^ (3.46 Ci) from a cyclotron was isolated from [^18^O]H_2_O by trapping it in a small MP1 fluoride trap and release cartridge that has been conditioned with water and air-dried. The [^18^F]F^-^ was then eluted with an acetonitrile/water mixture containing 20 mg of Kryptofix 222 and 5.0 mg of dipotassium phosphate trihydrate (K_2_HPO_4_.3H_2_O) into a conically shaped reaction vial previously purged with helium. The [^18^F]F^-^solution was evaporated under a small stream of helium at 100°C after which the residue was dried by azeotropic evaporation with anhydrous acetonitrile to ensure anhydrous reaction conditions were maintained for ^18^F labeling. After precursors **10** or **12** (2–3 mg, each) were added to the reaction vial, the resultant mixture was heated to 110°C for 15 min. After cooling to 30°C the reaction mixture was diluted with 4.4 mL of mobile phase (60% EtOH/H_2_O) and loaded onto a C-18 semi-preparative column (Macherey-Nagel C-18 250x10mm). The flow rate was increased from 0 to 6 mL/min over a 3 min time period. The 6 mL/min flow rate was maintained for 35 min during which the radioactive product was collected (28–31 min). The contents corresponding to the radioactive peak were diluted with 100 mL of distilled water and loaded onto a C-18 Sep-Pak® pre-conditioned with ethanol and water. The Sep-Pak was eluted by hand with 1 mL of 200 proof ethanol followed by 9 mL of saline. Qualitative control of the radioactive product was performed using radio-HPLC (C-18 column, Varian Dynamax, 4.6 × 250 mm, 30–75% gradient water to acetonitrile over 35 min, flow rate 1 mL/min) to confirm [^18^F]fluoride incorporation. The retention time was compared to that of the “cold” standard compound **11** (retention time = 20.4 min).

### CELL CULTURE AND TUMOR IMPLANTATION

Murine breast cancer cells, C57MG, 4T1, and 67NR were used as reported previously (Kobukai et al., 2011). Briefly, the cells were cultured and maintained in Dulbecco modified Eagle medium (Mediatech, Manassas, VA, USA) in the presence of 10% fetal calf serum (FCS; Invitrogen, Carlesbad, CA, USA), penicillin–streptomycin antibiotics (Mediatech), and 10 μg/mL insulin (Sigma–Aldrich, St Louis, MO, USA) at 37°C and 5% CO_2_ incubator.

The experimental protocol for animal imaging was approved by the Vanderbilt Medical Center Institutional Animal Care and Use Committee. Nude mice 6–8 weeks of age (*n* = 8, from Jackson Laboratory, Bar Harbor, ME, USA) were implanted subcutaneously under anesthesia (isoflurane mixed with 2% oxygen) with 1.0 × 10^6^ C57MG cells in the mammary fat pad. The progress of tumor growth was monitored via every-other-day measurement of tumor size and animal weight. When the tumors reached approximately 4 mm in diameter, *in vivo* PET imaging was performed.

### IC_50_ ASSAY

Various concentrations of the ^19^F-COX2 compound ranging from 0.1 μM to 0.3 nM were dispensed into designated wells within a 96-well microtiter plate at a final volume of 220 μL per well. Each well contained an assay buffer, heme, and ovine COX2 provided in Cayman’s colorimetric COX inhibitor screening assay kit. In addition to the tested probe, the assay condition was accompanied by background control wells and the 100% initial activity wells. Five minutes after incubation of all assay components at 25°C, an arachidonic acid substrate at a final concentration of 100 μM and the colorimetric co-substrate N,N,N′,N′-tetramethyl-p-phenylenediamine were added to each well. The plate was then incubated at 25°C for an additional 5 min before reading the absorbance at 590 nm using a plate reader. Absorbance of the duplicate assay of each well was averaged and subtracted from the 100% initial activity sample, after which it was divided by the 100% initial activity sample and multiplied by 100 to arrive at the percentage of inhibition.

### POSITRON EMISSION TOMOGRAPHY

Positron emission tomography imaging was performed using the microPET Focus 220 (Siemens Pre-clinical, Knoxville, TN, USA) in a static acquisition mode for 30 min at 60, 120, and 150 min after injection of ^18^F-COX2 probe **13** (150–200 μCi, 100–130 μL) into awake, non-fasted mice (*n* = 8) via the tail vein. To obtain whole-body scans, mice were placed in a supine position. The data were acquired in a 3-D mode with an axial span of approximately 8 cm. During the scanning, the animals were anesthetized using isoflurane and the temperature inside the scanner was maintained at 30°C using a pad connected to a circulating warm water bath. After PET imaging, a CT image was acquired using the microCAT II (Siemens Pre-clinical, Knoxville, TN, USA) using the same animal holder with the subjects maintained under anesthesia throughout, and then the mice were immediately euthanized upon completion of the CT scan. PET images were reconstructed using the iterative MAP reconstruction algorithm with 18 iterations and a beta smoothing value of 0.001 into 128 × 128 × 95 slices with a voxel size of 0.475 mm × 0.475 mm × 0.796 mm. The PET and CT images were co-registered using the imaging tool AMIDE (Loening and Gambhir, 2003).

### BIODISTRIBUTION

After the imaging session, the mice were euthanized and hearts, muscles, blood, livers, spleens, kidneys, stomachs, brains, intestines, tumors, and lungs were retrieved. The tissues were weighed and assessed for ^18^F radioactivity using a gamma counter (CRC-15W, Capintec, Ramsey, NJ, USA).

### WESTERN BLOT

Cells were washed twice with PBS, and lysed in ice-cold lysis buffer (50 mM Tris–HCl, pH7.4, 0.5% Triton X-100, 0.25% NP-40, 0.25% Na deoxycholate, 0.1% SDS, 150 mM NaCl, 1 mM EDTA), supplemented with complete anti-protease cocktail (Sigma). After removing nuclear and insoluble debris at 16,000*g* for 20 min, the supernatant designated as whole cell lysate (WCL) was saved. Protein concentrations were determined with Bradford method (Bio-Rad assay, Bio-Rad, Hercules, CA, USA). Thirty micrograms of WCL proteins were separated by 12% sodium dodecyl sulfate polyacrylamide gel electrophoresis (SDS-PAGE) and transferred onto polyvinylidene difluoride membranes (PVDF, Biorad). Membranes were blocked with 5% dry milk in Tris-buffered Saline with 0.1% Tween-20 (TBST) and immunoblotted overnight at 4°C with primary antibodies against COX2. β-Tubulin antibody (Santa Cruz) was used to blot same membrane for loading control. After washing with TBST three times, horse radish peroxidase (HRP) conjugated secondary antibodies were added for 1 h incubation. After wash with TBST twice and once with TBS, the protein bands were detected with an enhanced chemiluminescence (Pierce, Rockford, IL, USA) by exposure to films (Kodak) for 30 s. Band intensity was quantified by using NIH Image J software.

### REAL-TIME PCR

Total RNA was isolated and purified from cultured cells by using the Qiagen RNAeasy kit. RNA (2 mg) was reversibly transcribed by Superscript II (Invitrogen) with oligo-(dT) as primer to generate single stranded cDNA by following manufacturer recommended protocols. Quantitation of mRNA (cDNA) levels for COX2 was carried out by real-time PCR using S16P as internal controls. Real-time PCR primers were designed by web-based OligoPerfect^TM^ Designer (Invitrogen). The primer pairs used in PCR are forward 5′-CAGGAGAGAAGGAAATGGC-3′ and backward 5′-TGAGGAGAACAGATGGGATT-3′ to yield a 184nt product. Real-time PCR was carried out with the SYBR-green mixture from Bio-Rad in a final volume of 25 μL, with initial denaturation at 94°C for 3 min, followed by 45 cycles of denaturation at 94°C for 10 s, annealing and extension at 65°C for 1 min. PCR products were verified by acrylamide gel electrophoresis, melting curve analysis.

## RESULTS

### CHEMICAL SYNTHESIS AND CONFIRMATION OF THE ^18^F-COX2 PROBE

Starting with tropolone **1**, we synthesized three analogs of the precursor as shown in **Figure 1**. The advantage of working with azulene is that the reaction progress can be monitored via color changes. For example, evidence that compound **2** was converted to an aldehyde **3** using the Vilsmeier–Haack reaction resulted in a color change from blue to red. The thiazole ring was incorporated onto azulene in two steps. This included a hydrogenolysis reaction using triethylsilane in the presence of TFA to yield compound **6**, which is blue. Finally, we used Friedel–Crafts acylation to attach an aromatic ring to position 3 of the azulene. This reaction was completed using dichloroethane at room temperature for 30 min, which yielded the final product, which is brown. Under reaction conditions similar to those used with p-nitrobenzoyl chloride, 4-bromobenzoyl chloride provided an average yield of only 17% for the resultant Friedel–Crafts acylation product. The seemingly low yield can be attributed to the weak electron-withdrawing group. From an electronic perspective, we noted that the fluoro moiety is a much more favorable alternative than its bromo counterpart, as the fluoro derivative possesses greater electronegativity and is thus suitable for generating reactive electrophilic acylium ions. Notably, it is important to perform the Friedel–Crafts reaction as the last step since the nitro precursors will be reduced to amino groups under the reduction conditions. Compounds **10** and **12** were designed for [^18^F]fluoride labeling while compound **11** was used as a control to confirm the radiolabeling product and for specific activity analysis. All of the intermediates and products were characterized fully by ^1^H NMR and ^13^C NMR and mass spectrometry.

#### *^18^F-COX2* probe

We found this family of azulene compounds to be unstable under conventional ^18^F labeling conditions. After exhaustively analyzing every single reagent, solvent, and temperature involved in the labeling experiment, which included Kryptofix, Dimethyl sulfoxide (DMSO), DMF, acetonitrile, and potassium carbonate, we found by HPLC analysis that potassium carbonate was decomposing precursors **10** and **12** instantaneously at room temperature. This undesired chemical transformation was easily visualized since the color changed from brown to black when the precursors came into contact with potassium carbonate. Although we did not analyze the intermediates, this undesired reaction could be attributed to the acidic methylene protons between the azulene and thiazole ring, which may be sensitive to potassium carbonate. To overcome this problem, we decided to use a milder buffer such as dipotassium phosphate, which works perfectly for this purpose.

Although there was no sign of decomposition after we optimized the labeling conditions, the labeling of the bromo precursor **10** was sluggish. In contrast, we labeled successfully the nitro derivative **12**, albeit with low yield (3%, decay corrected) at EOS with >99% chemical and radiochemical purities and with a specific activity of 733 Ci/mmol.

### THE SPECIFICITY OF THE ^19^F-COX2 COMPOUND FOR THE COX2 ENZYME

**FIGURE 2:**
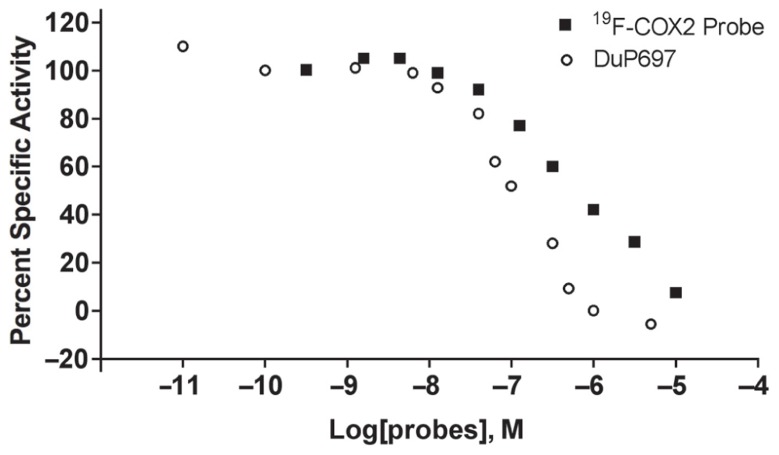
**^19^F-COX2 probe IC_50_ value determination using a colorimetric assay against DuP697, a known COX2 inhibitor in a 96-well plate format**. Data represent two independent experiments.

In addition to being synthesized for use in facilitating the confirmation of the ^18^F-labeled product, the cold compound **11** was also used to assess the IC_50_ value. The assay was performed using 10 duplicate concentrations in a range comparable to DuP697, a known COX2 inhibitor. As shown in **Figure 2**, the Hill slopes of the curves that represent ^19^F-COX2 and DuP697 are -0.62 and -1.0, respectively; suggesting the specificity of the synthesized PET probe for COX2. After taking the background signal into account, the IC_50_ value of the ^19^F-COX2 compound was 661 nM.

### COX2 IS OVEREXPRESSED IN C57MG BREAST CANCER CELLS

**FIGURE 3:**
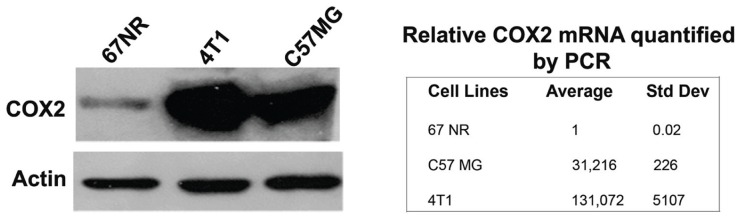
**Analysis of COX2 expression and quantity in murine breast cancer cells**. Western blot analysis was performed to verify the presence and relative intensity of COX2 in C57GM cells compared to other cells. β-actin served as a loading control (left). RT-PCR data were used to quantify the level of COX2 expression after normalization.

To confirm and quantify COX2 expression in the C57MG cell line, we selected two other cells, 4T1 and 67NR, which are also murine breast cancer cell lines. It has been demonstrated previously that the 4T1 (Harmey et al., 2002) and 67NR cells (Nagler et al., 2011) were positive and negative, respectively, for COX2. As shown in **Figure 3**, Western blot analysis on cell lysate indicated a very low level of COX2 in 67NR cells. In contrast, C57MG possesses a high constitutive level of COX2. Furthermore, real-time PCR data demonstrated that COX2 was expressed at a rate approximately 31-fold higher in C57MG cells compared to 67NR.

### *IN VIVO* IMAGING OF COX2 IN TUMOR MOUSE MODEL AND BIODISTRIBUTION

**FIGURE 4:**
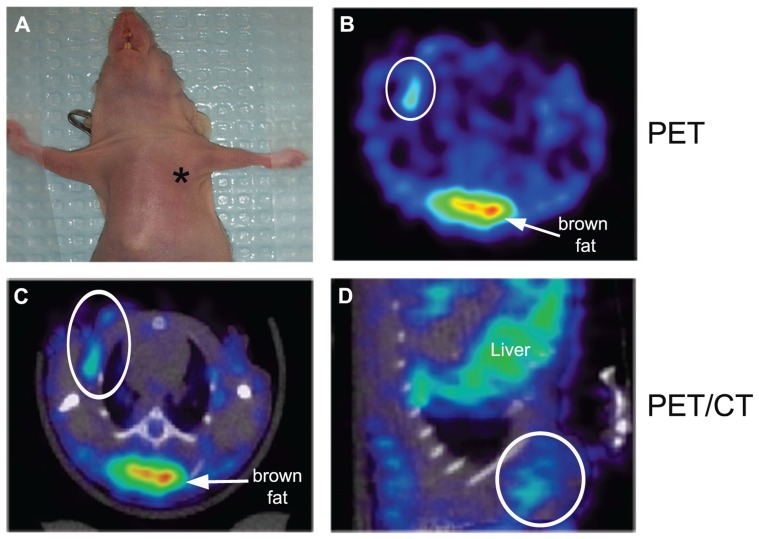
***In vivo* microPET imaging of COX2 in a tumor-bearing mouse model**. At the time of imaging, tumor size was approximately 4 mm in diameter (*, tumor) **(A)**. Representative PET image of an axial section showing tumor uptake of ^18^F-COX2 probe (white circle) **(B)**. Fused PET/CT axial image **(C)**. Fused PET/CT sagittal view of the tumor **(D)**.

**FIGURE 5:**
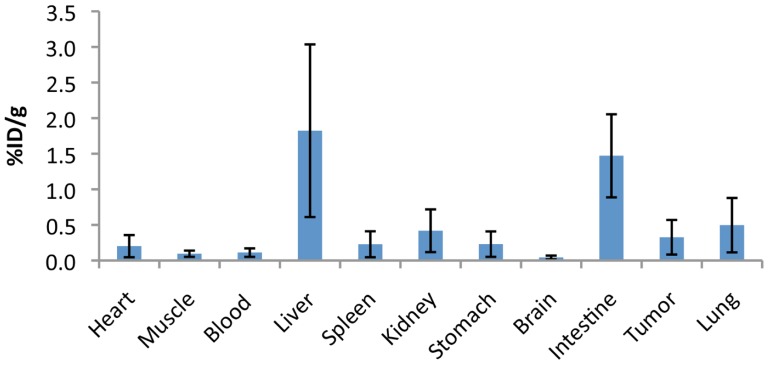
**Uptake (% injection dose/g tissue) of the ^18^F-COX2 probe 13 in non-fasted tumor-bearing mice (*n* = 3)**.

To assess the specificity of the probes for the detection of COX2 expression, we performed *in vivo* PET imaging of non-fasted mice in which C57MG tumors had been implanted on the mammary fat pads. We monitored the distribution of the probe in breast cancer at several times after the intravenous bolus injection. The optimal emission data were collected during a static, whole-body scan 150 min after administration of the probe. PET and PET/CT images showed accumulation and retention of the ^18^F-COX2 probe **13** and that significant accumulation in the tumor resulted in high signal intensity compared to the background (*p* < 0.05; **Figure 4**). The PET data corroborates with Western blot and RT-PCR analysis. We also observed a predominant hepatic uptake of the probe. That outcome is reasonably understandable since lipophilic compounds tend to possess a strong affinity for the liver. In addition, the high liver-bowel activity observed in this study suggests the possibility of hepatobiliary excretion. The probe exhibited negligible signal in the bone, thus eliminating the notion of *in vivo* defluorination. **Figure 5** shows the probe’s biodistribution in non-fasted tumor-bearing mice (*n* = 3) at 150 min post injection. The data shows that the probe accumulated in the tumor; however, the highest uptake was detected in the liver, followed by the intestine. It is very likely that the high activity observed in the intestine can be attributed partially to the stool residuals.

### STATISTICS

Student’s *t*-test was used to evaluate statistical differences between samples. Significant differences were considered as *p* < 0.05.

## DISCUSSION

The goal of this work is to design, synthesize and test a novel class of azulene-based probes with which to image COX2 in cancer. Although synthesis of this class of COX2 inhibitors has been reported in the past (Tomiyama et al., 1999), conversion from an inhibitor to a contrast agent requires an entirely different chemistry. This is because the chemistry used originally is unsuitable for producing the nitro precursor **12**. Conversion of a nitrile derivative into thioamide using hydrogen sulfide, shown by Tomiyama et al. (1999), concomitantly reduces the nitro to an amine. Another disadvantage of constructing the thioamide directly from the azulene ring is that the process requires many steps of synthesis, and failure in any single step in the link will affect the whole scheme. In this project, we utilized a convergent synthesis strategy wherein the three major rings of the compound were either synthesized or acquired from a diverse library of analogs commercially. These were then assembled into the desired product using simple chemistry. Thanks to this approach, we shortened the synthesis by three or four steps. In addition, the approach enables the potential generation of a library of compounds with novel functional groups that offer untapped bioisosteres.

Another innovative approach of this work lies in the ^18^F labeling process. To our knowledge, currently, there are no reported data showing an alternative buffer to the conventional use of potassium carbonate. We hypothesized that the role played by potassium is that of serving as a counter-ion for the [^18^F]fluoride and as such it can be displaced by a similar cation. However, for these precursors or any basic sensitive compounds, weaker bases such as dipotassium phosphate should be used as an alternative since their pH is nearly neutral. Since we have not performed this sort of experiment on other types of compounds, we cannot extrapolate the reason why the specific activity of final product is low. More work is under progress to improve the specific activity of compound **13**. One approach in that direction is to use high-grade dipotassium phosphate to ensure the elimination of trace fluoride in the labeling process. Nevertheless, in view of our recent findings and in light of the high number of basic sensitive precursors that failed in PET labeling, it is appropriate to hope that this finding can provide far-reaching applications for other compounds.

*In vivo* PET imaging demonstrated that there was no defluorination of the probe *in vivo* even 2.5 h post injection of the radioligand. To our knowledge, this is the first COX2 PET radioligand demonstrating such high stability *in vivo*. However, as the scope of this article was to report the chemical development of the probe, future studies will be needed to fully characterize this radioligand *in vivo* which include blood sampling and kinetic modeling as well as displacement studies. In addition, other important issues still need further evaluation. For example, we do not have information regarding tumor uptake between fasted and non-fasted mice. Although there is no systematic or mechanism that explains the difference between these two groups of study, Fueger et al. (2006) reported that in fasted mice, tumor uptake increased fourfold while tumor-to-organ ratios increased up to 17-fold compared to the non-fasted counterparts. Currently, work is in progress in our group to address this issue. Furthermore, *in vivo* blocking studies using cold compound **11** or COX2 inhibitors would be ideal to further confirm the specificity of this PET probe.

Data obtained in this work suggest that this probe not only has the potential to detect inflammation, but it can also be used to detect the early onset of cancer. Furthermore, this targeted imaging approach is applicable for the assessment of tumor response during chemotherapy. Another application for the *in vivo* imaging of COX2 lies in cell therapy. Muthuswamy et al. (2010) showed that COX2 impairs the ability of dendritic cells (DCs) to attract naïve T cells. One of the mechanisms involved is that COX2 inhibits the ability of DCs to produce CCL19. In another study, Harizi et al. (2002) showed that COX2 induced PGE2 enhances the production of endogenous IL-10, which downregulates DC functions. By using COX2 inhibitors to attenuate the expression of IL-10 and the concomitant restoration of IL-12 production by DCs, Stolina et al. (2000) demonstrated that the COX2 inhibitor can modulate and be used beneficially as an adjuvant strategy in cancer therapy. Altogether, we believe that non-invasive imaging of COX2 with this probe in breast cancer would provide valuable insight into the tumor microenvironment.

In conclusion, we have demonstrated an innovative synthetic approach to the development of a novel class of ^18^F-COX2 contrast agents. In addition, we reported on the optimized labeling conditions that can be applied to any base-sensitive PET precursors. The chemistry we utilized is reproducible and scalable, and each step of the syntheses described in this work has been repeated and characterized more than 30 times by NMR and mass spectrometry. Most importantly, small animal PET imaging data suggest the specificity of the probe for COX2. In general, it seems reasonably certain that this class of azulene-based agents deserves further evaluation, as *in vivo* imaging of COX2 will offer significant insights into the implication of this enzyme in the inflammation–dysplasia–cancer matrix.

## Conflict of Interest Statement

The authors declare that the research was conducted in the absence of any commercial or financial relationships that could be construed as a potential conflict of interest.
